# Plasma Cytokines and Birth Weight as Biomarkers of Vaccine-Induced Humoral Responses in Piglets

**DOI:** 10.3389/fvets.2022.922992

**Published:** 2022-07-13

**Authors:** Sean Lipsit, Antonio Facciuolo, Erin Scruten, Philip Griebel, Scott Napper

**Affiliations:** ^1^Vaccine and Infectious Disease Organization (VIDO), University of Saskatchewan, Saskatoon, SK, Canada; ^2^Department of Biochemistry, Microbiology and Immunology, University of Saskatchewan, Saskatoon, SK, Canada; ^3^School of Public Health, University of Saskatchewan, Saskatoon, SK, Canada

**Keywords:** biomarker, pro-inflammatory cytokines, interferon-gamma, vaccine response, birth weight

## Abstract

Failure to mount an effective immune response to vaccination leaves individuals at risk for infection and can compromise herd immunity. Vaccine unresponsiveness can range from poor responses “low responders” to a failure to seroconvert “non-responders.” Biomarkers of vaccine unresponsiveness, particularly those measured at the time of vaccination, could facilitate more strategic vaccination programs. We previously reported that pro-inflammatory cytokine signaling within peripheral blood mononuclear cells, elevated plasma interferon-gamma (IFNγ), and low birth weight correlated with vaccine-induced serum IgG titers in piglets that were below the threshold of detectable seroconversion (vaccine non-responders). These observations suggested that plasma IFNγ concentration and birth weight might serve as pre-vaccination biomarkers of vaccine unresponsiveness. To test this hypothesis, piglets (*n* = 67) from a different production facility were vaccinated with the same commercial *Mycoplasma hyopneumoniae* bacterin (RespiSure-One) to determine if there was a consistent and significant association between vaccine-induced serum IgG titers and either plasma cytokine concentrations or birth weight. All piglets seroconverted following vaccination with significantly less variability in vaccine-induced serum IgG titers than observed in the previous vaccine trial. Piglets exhibited highly variable birth weights and plasma cytokine concentrations prior to vaccination, but there were no significant associations (*p* > 0.05) between these variables and vaccine-induced serum IgG titers. There were significant (*p* < 0.001) differences in plasma IFNγ concentrations among individual litters (*n* = 6), and plasma IFNγ concentrations decreased in all pigs from birth to 63-days of age. One of the six litters (*n* = 11 piglets) exhibited significantly elevated plasma IFNγ concentrations during the first 3 weeks of life (*p* < 0.001) and at the time of vaccination (*p* < 0.01). This litter, however, had similar vaccine-induced serum IgG titers when compared to the other piglets in this study. Collectively the two studies indicate that while plasma cytokines and birth weight can be associated with vaccine non-responsiveness, their temporal and individual variation, as well as the complexity of the vaccine responsiveness phenotype, make them inconsistent biomarkers for predicting the less extreme phenotype of vaccine low responders.

## Introduction

Infectious diseases are among the greatest sources of economic loss to livestock producers (1, 2). While vaccines remain an essential tool for managing infectious diseases, individual variability within heterogeneous populations can limit the extent to which vaccines induce immune responses. Vaccination of pig populations can result in a wide-range of immune responses with some animals not developing vaccine-specific immunity (3–5). As a result, individuals failing to respond “non-responders,” or responding poorly “low responders,” remain at risk for infection and can compromise disease protection afforded through herd immunity (6, 7). Vaccine unresponsive individuals may also be detrimental to herd health when management decisions are made based on the assumption that vaccination equates to protection. The ability to identify unresponsive individuals at the time of vaccination would enable more informed decisions to protect animal health at individual and population levels.

Identifying vaccine unresponsive individuals in a population usually requires quantifying a surrogate measure of immunity, such as antigen-specific antibody titers or T-cell responses, in the weeks following immunization (8). A limitation of this approach is that it does not address the period during which vaccine unresponsive individuals remain susceptible to infection while co-mingling with the herd. Biomarkers that predict vaccine-induced immune responses at the time of vaccination would enable the immediate identification of vaccine unresponsive individuals and facilitate strategic management decisions to protect herd health, such as revaccination, physical isolation, or culling (9, 10). In addition, biomarkers that provide information regarding the molecular basis of vaccine unresponsiveness could improve vaccine formulation and delivery strategies to maximize the percentage of vaccine responders (11).

Identification of vaccine response biomarkers has typically focused on delineating the host factors affecting vaccine responsiveness in heterogeneous, outbred populations. For example, within humans, genome-wide association studies have identified polymorphisms within coding regions of antigenic proteins, cell signaling intermediates, and cytokines associated with various aspects of immune function associated with vaccine responsiveness (12–14). In addition to genetic factors, chronic conditions such as aging (15, 16) and obesity (17, 18), as well as acute conditions, such as inflammation (19), infection (20), or microbiome disruption (21) can impact an individual's immune response to vaccination. Molecular mechanisms influencing vaccine responsiveness have been characterized thoroughly using systems biology approaches both prior to (22–24), and following, immunization (25, 26). In livestock species, heritability estimates and genetic polymorphisms associated with vaccine responsiveness have been major areas of focus (4, 5, 27). Furthermore, transcriptional analysis of host responses following vaccination against pathogens such as foot-and-mouth-disease virus (28), tetanus toxoid (3), and *Mycoplasma hyopneumoniae* (*M. hyopneumoniae*) (29, 30) are of interest. While these efforts have provided valuable insights into the molecular and cellular events associated with both successful and unsuccessful vaccination, they have not provided reliable biomarkers that predict immune responses prior to vaccination.

Recently, our group delineated signaling events associated with vaccine unresponsiveness in piglets immunized with the commercial intramuscular vaccine, RespiSure-One (31). In that study, we analyzed the phosphorylation-mediated signal transduction (kinome) of peripheral blood mononuclear cells (PBMCs) collected from individual pigs at each extreme of vaccine response prior to vaccination. Based on the recommended seroconversion threshold of *M. hyopneumoniae*-specific serum antibody responses with a post-vaccination S/P ratio >0.4 (approximately a Log_2_ endpoint titer of 9.7) the low responders of this trial, with a titer range = 5.8–7.7, were seronegative, or vaccine non-responders. This analysis revealed differential pro-inflammatory signaling when comparing high and low vaccine responders. Subsequent analysis of plasma cytokines confirmed higher concentrations of interferon-gamma (IFNγ) and interleukin-1 beta (IL-1β) at the time of vaccination in vaccine non-responders as compared to high responders. These data suggested that a pro-inflammatory environment at the time of vaccination may hinder vaccine-induced immune responses. Additionally, the vaccine non-responders had consistently lower body weight prior to vaccination than high responders, suggesting delayed piglet growth and development influenced adaptive immune responses to vaccines (31). These data, along with other studies correlating pro-inflammatory serum cytokines (19, 32), inflammatory gene expression (24, 33), activated innate immune cells (34), and neonatal host factors (35, 36) with vaccine-specific responses support the hypothesis that pro-inflammatory events within the host at the time of vaccination and low birth weight can negatively impact vaccine-induced antibody responses.

The objective of this study was to further investigate the relationships between vaccine-induced IgG titers, pro-inflammatory plasma cytokine concentrations prior to vaccination, and birthweight. The current study was performed with an independent population (*n* = 67) of piglets raised in a different production facility than piglets in the previous study. Using vaccine-induced serum IgG titers as the metric for vaccine responsiveness, we report that elevated plasma cytokines and low birth weight are not consistent biomarkers of vaccine responsiveness in piglets vaccinated with RespiSure-One. All piglets in the current study responded to vaccination with detectable antibody titers despite significant variation in plasma IFNγ and IL-1β concentrations at multiple time-points prior to vaccination. Variations in plasma cytokine concentration were litter-specific and could be attributed to piglets' genetic/maternal background. Additionally, piglets of high and low birth weights responded similarly to vaccination generating a seropositive response after two vaccinations. These findings confirm that substantial variations in plasma IFNγ and IL-1β occur in young piglets and demonstrate that plasma cytokine concentrations or body weight do not always correlate impaired vaccine responsiveness.

## Materials and Methods

### Animal Care and Vaccination

The experimental protocol (AUP20190084) was approved by the University of Saskatchewan Animal Care and Use Committee in accordance with the Canadian Council on Animal Care guidelines. Animal description, vaccination schedule, and sample collection schedule was consistent with our initial investigation (named “Trial 2015”) as described previously (31). Specifically, piglets were monitored for changes in weight gain, behavior, and clinical signs of infection, physical injury, or trauma under the attention and care of licensed veterinarians at Prairie Swine Center (Saskatoon, SK, Canada). Routine monitoring and clinical assessment of piglets throughout Trial 2020 did not identify clinical signs of infection, physical injury, or trauma. No specific data on general health is included as the facility veterinarians and animal care team reported no clinical signs or health concerns from animals within this study cohort to warrant a full diagnostic evaluation.

Six Camborough Plus sows (parity = 0–3) bore litters (*n* = 8–14 piglets/litter) for a total of 67 piglets (37 males; 30 females) enrolled in the current study (named “Trial 2020”). Piglets were nursed with littermates until weaning at 24 ± 1 days of age (D24). Piglets were vaccinated intramuscularly with RespiSure-One (1 mL/dose; Zoetis, USA) at 27 ± 1 days of age (D28) and given a booster vaccine at 51 ± 1 days of age. The trial was terminated when piglets were 62 ± 1 days of age (D63). Body weight was measured at birth (D0), D7, D14, D21, D24, and D63.

### Serum and Plasma Collection

Starting at birth (D0), whole blood was collected from the jugular vein into Vacutainer K_2_EDTA-coated and serum separation tubes (Becton Dickinson) for the collection of plasma and serum, respectively. Samples were collected weekly within the same 2-h period to minimize effects due to circadian rhythms. Plasma was collected from piglets on D0, D7, D14, D21, D28, and D63. Serum was collected on D28 (prior to primary vaccination) and D63 (11-days after booster vaccination). Serum separation tubes were incubated for 30 min at room temperature. Serum and plasma were centrifuged at 2,000 × g for 20 min, 4°C, without break. Aliquots of serum and plasma were stored at −80°C.

### Serum *Mycoplasma hyopneumoniae* IgG ELISA

One mL serum collected from each piglet (Trial 2020) collected on D28 and D63 was shipped to Prairie Diagnostic Services (Saskatoon, SK, Canada) where *M. hyopneumoniae*-specific IgG titers were quantified using an IDEXX *Mycoplasma hyopneumoniae* Antibody Test Kit ELISA (IDEXX Laboratories, Inc.).

To determine S/P ratios ($\frac{sample\;A_{650}\;-negative\;A_{650}}{positive\;A_{650}-negative\;A_{650}}$), serum were diluted 1:40 and A_650_ absorbance were recorded. Serum was considered seronegative when S/P < 0.3 and seropositive if S/P > 0.4, as per the manufacturer's instructions.

To quantify serum *M. hyopneumoniae*-specific IgG titers for each piglet on D63, a modified endpoint titration ELISA was used (IDEXX *M. hyo* Ab Test Kit ELISA). D63 serum were serially diluted 4-fold beginning with an initial 1:40 dilution. ELISA reactions were quantified using A_650_ absorbance. Endpoint titers were calculated by subtracting the negative control A_650_ absorbance and taking the reciprocal of the highest dilution with an A_650_ absorbance greater than the mean of the negative control. Positive and negative controls were porcine anti-*M. hyopneumoniae* serum (IDEXX Laboratories, Inc.), and porcine serum non-reactive to *M. hyopneumoniae* (IDEXX Laboratories, Inc.), respectively. Endpoint titration ELISAs were completed by Biovet (Saint-Hyacinthe, QC, Canada).

### Serum *Mycoplasma hyopneumoniae* IgG ELISA

One mL serum collected from each piglet (Trial 2020) collected on D28 and D63 was shipped to Prairie Diagnostic Services (Saskatoon, SK, Canada) where *M. hyopneumoniae*-specific IgG titers were quantified using an IDEXX *Mycoplasma hyopneumoniae* Antibody Test Kit ELISA (IDEXX Laboratories, Inc.). Serum were diluted 1:40, and S/P ratios ($\frac{sample\;A_{650}\;-negative\;A_{650}}{positive\;A_{650}-negative\;A_{650}}$) were calculated. Serum was considered seronegative when the S/P ratio at a 1:40 dilution was S/P < 0.3 and seropositive if S/P > 0.4, as per the manufacturer's instructions.

Serum *M. hyopneumoniae*-specific IgG titers were determined for each piglet on D63 and quantified using a modified endpoint titration ELISA (IDEXX *M. hyo* Ab Test Kit ELISA). D63 serum were serially diluted 4-fold beginning with an initial 1:40 dilution. ELISA reactions were quantified using A_650_ absorbance. Endpoint titers were calculated by subtracting the negative control A_650_ absorbance and taking the reciprocal of the highest dilution with an A_650_ absorbance greater than the mean of the negative control. Positive and negative controls were porcine anti-*M. hyopneumoniae* serum (IDEXX Laboratories, Inc.), and porcine serum non-reactive to *M. hyopneumoniae* (IDEXX Laboratories, Inc.), respectively. Endpoint titration ELISAs were completed by Biovet (Saint-Hyacinthe, QC, Canada).

In this study, *M. hyopneumoniae-*specific antibodies were not measured for sows, but the facility housing the sows and piglets had not used *M. hyopneumoniae* vaccines or diagnosed an animal with *M. hyopneumoniae* infection for 5 years prior to this study. All the piglets were seronegative for (S/P < 0.3) for *M. hyopneumoniae* antibodies prior to vaccination at 28-days of age (D28). We therefore assume that vaccine-induced *M. hyopneumoniae*-specific maternal antibodies were not transferred to the piglets used in this study and the antibodies to *M. hyopneumoniae* are a direct consequence of vaccination of the piglets.

The calculation of serum *M. hyopneumoniae*-specific antibody titers for Trial 2015 were described previously (31). Consistent with that investigation, for the current study plasma was collected from high (*n* = 6) and low (*n* = 6) responders on D28 was centrifuged (2,000 × g, 30 min) and shipped to Prairie Diagnostic Services (Saskatoon, SK, Canada), and S/P ratios were calculated (IDEXX Laboratories, Inc.).

### Porcine-Specific Multiplex Cytokine Analysis

The porcine-specific multiplex assay was conducted as described previously with the following modifications (31). Briefly, recombinant porcine cytokine standards for IFNγ (2,000 pg/mL; VIDO) and IL-1β (5,000 pg/mL; R&D Systems; cat. 681PI010) were diluted 1:3 in New Zealand pig serum to account for serum inhibitory effects, while samples were diluted 1:3 in diluent (PBS, 1% New Zealand pig serum, 0.05% sodium-azide). Porcine-specific antibodies for IFNγ (Fisher Scientific; cat. ENMP700, Clone P2F6) and IL-1β (R&D Systems; cat. MAB6811, Clone 77724) were conjugated to individual BioPlex Max-Plex C magnetic beads (Bio-Rad Laboratories Inc.) based on the method described by Lawson et al. (37). Anti-porcine antibodies specific to IFNγ (Fisher Scientific; polyclonal antibody cat. PIPP700) and IL-1β (R&D Systems; biotinylated antibody cat. BAF681) were used in to detect porcine cytokines. All samples were analyzed in triplicate. Technical replicates with a coefficient of variation (CoV) <30% were averaged for a final sample concentration. Technical replicates with a CoV >30% had the outlying (SD ± 1) replicate removed and the remaining two replicates were averaged. Samples with technical replicates below the lower limit of quantification were not included in the average result.

### Statistical Analysis

All data analysis and visualization were performed using GraphPad Prism version 9.0 (GraphPad Software, San Diego, California USA). The correlation matrix (**Figure 4A**) was created in R using the package “corrplot” (38). The Log_2_-transformed *M. hyopneumoniae*-specific IgG titer (serum IgG titers) from Trial 2015 was determined to be normally distributed [one-sample Kolmogorov–Smirnov (KS) test, *p* > 0.1]. Serum IgG titers from Trial 2020 were not normally distributed (one-sample KS-test, *p* < 0.001), however, the sample size (*n* = 67) was large, samples were measured independently, and the mean (11.6) approximated the median (11.8), so the data was treated as being normally distributed. A two-sample KS-test was used to determine differences in distributions of serum IgG titers between Trial 2015 and Trial 2020. A two-tailed, unpaired Student's *t*-test with Welch's correction was used to determine differences of serum IgG titers between Trial 2015 HR and Trial 2020 HR, as well as between Trial 2015 LR and Trial 2020 LR (*F*-test of equality of variances, *p* < 0.05). Plasma cytokine concentrations of piglets from Trial 2020 were not normally distributed on individual days (one-sample KS test, *p* < 0.1). Therefore, a Kruskal-Wallis one-way ANOVA with Dunn's multiple comparisons was used to detect differences in IFNγ and IL-1β concentrations among days. However, plasma cytokine concentrations were normally distributed when grouped by litter (one-sample KS test, *p* > 0.1). A two-way mixed-effects ANOVA with a Geisser-Greenhouse correction was conducted using the factors “Litter” and “Day.” Tukey's multiple comparisons were conducted between litters on each day due to the effect of “Litter x Day” for plasma IFNγ (*F*_25, 270_ = 41.4, *p* < 0.001) and IL-1β (*F*_25, 298_ = 9.06, *p* < 0.001). A one-way ANOVA was conducted to determine differences in serum IgG titers between litters. A Spearman Rank Correlation was conducted to correlate serum IgG titer and plasma cytokine concentrations. Body weight of all piglets at birth (D0), weaning (D24), and at the end of the trial (D63) were determined to be normally distributed (Kolmogorov–Smirnov test, *p* > 0.1). A two-tailed unpaired Student's *t*-test with Welch's correction was conducted to analyze differences in body weight and average daily gain (kg/day) between HR and LR, and to analyze differences in serum IgG titer between the highest and lowest birth weight piglets. *P*-values < 0.05 were considered statistically significant and *p*-values at 0.05 < *p* < 0.1 were considered a statistical trend.

## Results

### Variability of Vaccine-Induced Serum *Mycoplasma hyopneumoniae-*Specific IgG Titers

Piglets (*n* = 67) born from multiple litters (6 litters, 8–14 piglets/litter) were vaccinated (RespiSure-One) at 28 days of age (D28, 4 days post-weaning) and boosted at 52 days of age. *Mycoplasma hyopneumoniae* (*M. hyopneumoniae*)-specific serum IgG titers were quantified prior to vaccination (D28) and 11-days following booster vaccination at 63 days of age (D63) using an IDEXX *Mycoplasma hyopneumoniae* Antibody Test Kit (IDEXX Laboratories, Inc.). All piglets within the current trial (“Trial 2020”) were seronegative (S/P < 0.3) on D28 and all piglets were seroconverted (S/P > 0.4) on D63.

**Figure 1:**
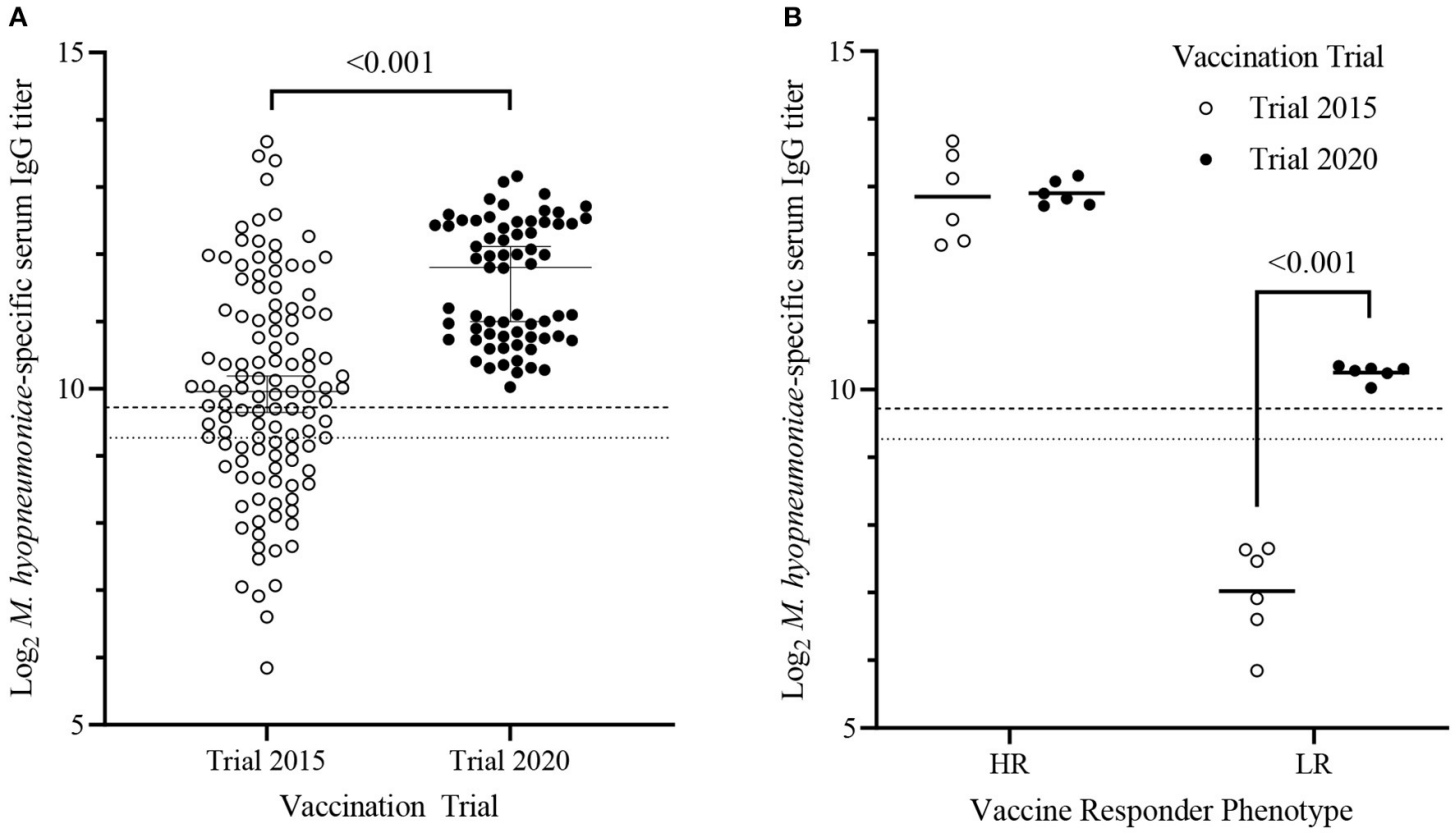
*Mycoplasma hyopneumoniae*-specific serum IgG titers of piglets from Trial 2015 and Trial 2020 11-days following RespiSure-One booster vaccination. **(A)** Median (±95% confidence interval) Log_2_ *M. hyopneumoniae*–specific serum IgG titers of piglets from Trial 2015 (*n* = 117; open circles) and Trial 2020 (*n* = 67; solid circles). Differences were determined using a 2-sample Kolmogorov–Smirnov test. **(B)** Mean serum *M. hyopneumoniae*–specific IgG titers of the high (HR) and low (LR) responders within Trial 2015 (*n* = 6/cohort; open circles) and Trial 2020 (*n* = 6/cohort; closed circles). The horizontal bar represents the group mean. Dashed and dotted lines represent the threshold for seropositive and seronegative cutoffs, respectively. Differences were determined using a two-tailed, unpaired Student's *t*-test with Welch's correction.

To quantify vaccine-induced antibody responses, D63 serum from each piglet were serially diluted 5-fold and endpoint serum IgG titers were calculated. There was an 8-fold difference between the highest and lowest Log_2_ serum IgG titers [titer range = 10.0–13.2; median (95% CI) = 11.8 (11.0–12.1)] within the trial population. No sex-dependent (Mann-Whitney *U*-test, *p* = 0.13) effect on serum IgG titers was observed. Serum samples from piglets within Trial 2020 were collected in two batches (3 litters/batch) on different days, and there was no batch-dependent effect (Mann-Whitney *U*-test, *p* = 0.35). There was no significant difference (*p* > 0.8) in serum IgG titers on D63 among any of the litters. Compared to our previous trial (“Trial 2015”) [log_2_ titer range = 5.85–13.67; median (95% CI) = 9.96 (9.65–10.2)], piglets from Trial 2020 had higher (*p* < 0.001) median serum IgG titers with less variation in titers (Figure 1A) (31).

In our previous study, we stratified piglets in the 10 and 90th percentile of serum IgG titers into low (LR; *n* = 6) and high (HR; *n* = 6) responders, respectively (31). HR and LR from Trial 2015 were confirmed to be seronegative (S/P < 0.3) on D28. The same criterion was used to stratify piglets in Trial 2020 into LR (*n* = 6) and HR (*n* = 6). There was no difference (*p* = 0.89) when comparing mean serum IgG titers between HR of Trial 2020 (12.9 ± 0.18, mean ± SD) and HR of Trial 2015 (12.9 ± 0.64, mean ± SD). In contrast, Trial 2020 LR were seropositive (S/P > 0.4) and had significantly higher (*p* < 0.001) mean serum IgG titers (10.3 ± 0.11; mean ± SD) than the seronegative Trial 2015 LR (5.85 ± 0.71; mean ± SD) (Figure 1B). Thus, the Trial 2020 LR cohort did not phenotypically represent seronegative non-responders. Trial 2020 LR more appropriately represent seropositive vaccine responders with lower serum IgG titers than the HR. However, the Trial 2020 LR had significantly lower serum IgG titers than the Trial 2020 HR and were used to test the previously identified associations between vaccine-induced antibody responses and either plasma cytokines or birth weight (31).

### Relation Between Vaccine Responses and Pro-inflammatory Cytokines

**Figure 2:**
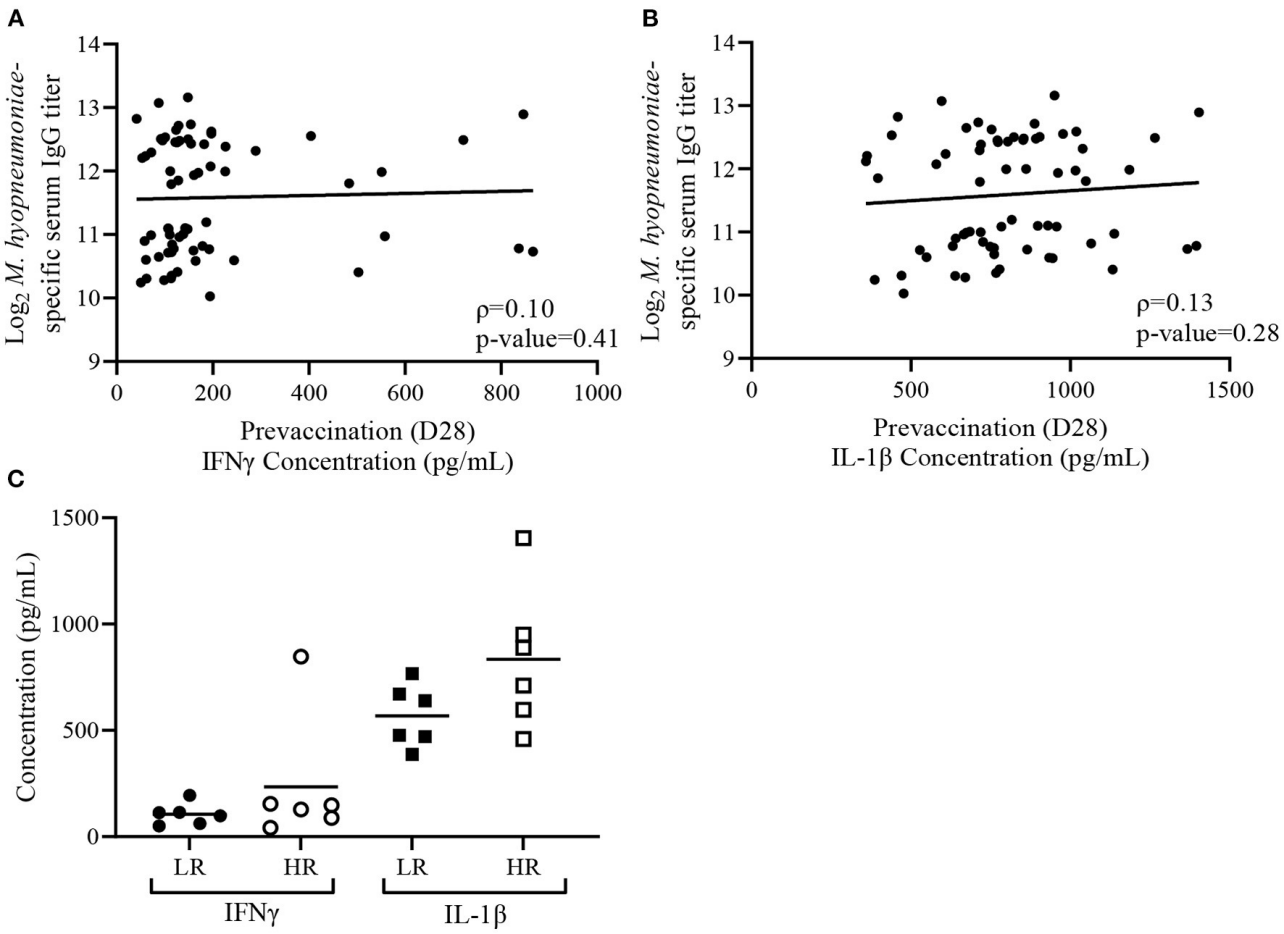
Variability in plasma cytokine concentrations does not associate with *Mycoplasma hyopneumoniae*-specific IgG responses in the Trial 2020. **(A)** Spearman correlation analysis of D63 *M. hyopneumoniae*-specific serum IgG titers with D28 plasma interferon-gamma (IFNγ) and **(B)** D28 plasma interleukin 1-beta (IL-1β) concentrations from Trial 2020 (*n* = 67). Lines represents best-fit lines. **(C)** Mean plasma concentrations of IFNγ (circles) and IL-1β (squares) in low (LR; *n* = 6; closed symbol) and high (HR; *n* = 6; open symbol) responders from Trial 2020. Differences determined using a Student's *t*-test with Welch's correction.

Previously, we observed that plasma IFNγ and IL-1β levels prior to vaccination (D28) were negatively associated with *M. hyopneumoniae*-specific IgG titers in age-matched piglets 35 days post-vaccination (31). To further test the relationship between D28 plasma cytokine concentrations and vaccine-induced antibody responses, correlation and comparative analyses of D28 plasma IFNγ and IL-1β concentrations with serum IgG titers were conducted using piglets from Trial 2020. Within Trial 2020, D28 plasma IFNγ levels prior to vaccination did not show a significant (ρ = 0.10, *p*-value = 0.41) Spearman correlation with D63 post-vaccination serum IgG titers among all piglets (*n* = 67) (Figure 2A). Similarly, D28 plasma IL-1β levels prior to vaccination and serum IgG titers at D63 were not correlated (ρ = 0.13, *p*-value = 0.28) among all piglets within Trial 2020 (Figure 2B). Limiting the analysis to only the HR (*n* = 6) and LR (*n* = 6) from Trial 2020 revealed no differences in either plasma IFNγ (*p* = 0.34) or IL-1β (*p* = 0.12) concentrations prior to vaccination (Figure 2C). Altogether, no significant association between pre-vaccination plasma cytokines concentrations and vaccine-induced serum IgG titers were detected with piglets in Trial 2020.

### Temporal Analysis of Plasma Cytokines

**Figure 3:**
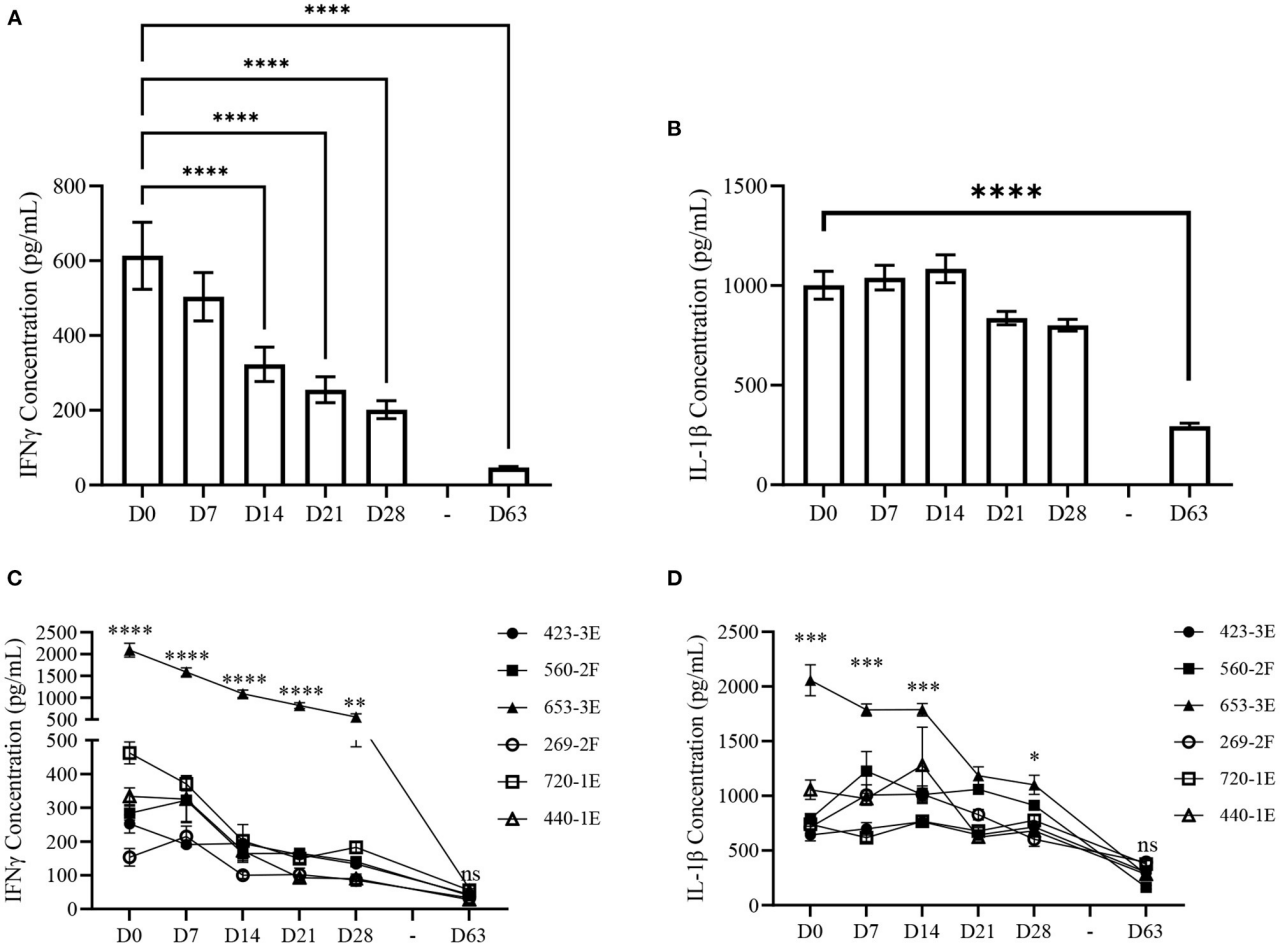
Plasma cytokine concentrations of Trial 2020 piglets from birth (D0) to 63-days of age (D63) at weekly intervals. **(A)** Mean (±SEM) plasma interferon-gamma (IFNγ) and **(B)** interleukin 1-beta (IL-1β) concentrations measured weekly from piglets within Trial 2020 (*n* = 67) from birth (D0) until vaccination (D28) and at 63-days of age (D63). Differences determined using a Kruskal-Wallis one-way ANOVA with Dunn's multiple comparisons. **(C)** Mean (±SEM) plasma IFNγ and **(D)** IL-1β concentrations measured weekly from D0 to D28, and at D63, of Trial 2020 (*n* = 67) grouped by the factor “Litter” (423–3E, *n* = 14; 560–2F, *n* = 12; 653–3E, *n* = 11; 269–2F, *n* = 8; 720–1E, *n* = 12; 440–1E, *n* = 10). Differences determined using a two-way mixed effects ANOVA with Tukey's multiple comparisons. Asterisks designate differences in mean cytokine concentration between litters. Only *p*-values representing the differences across all multiple comparisons between litters are shown. ^n.s.^*p* > 0.05; *p < 0.05; **p < 0.01; ***p < 0.005; ****p < 0.001.

Our previous trial (Trial 2015) revealed that prior to vaccination piglets with lower vaccine-induced antibody responses had elevated levels of plasma cytokines IFNγ and IL-1β compared to age-matched piglets with high vaccine-induced antibody responses (31). However, these plasma cytokines were measured only at 28-days of age (D28) and it was not determined whether cytokine concentrations were temporally stable prior to vaccination. Within piglets from Trial 2020, plasma IFNγ and IL-1β was quantified at 7-day intervals beginning at birth (D0) up to D28 and ending at D63 to determine if IFNγ and IL-1β concentrations remained constant throughout the timeframe during which we measured vaccine responsiveness (D0–D63). Plasma IFNγ (Supplementary Table 1) and IL-1β (Supplementary Table 2) concentrations of individual piglets for each time point have been appended. There was a significant (*p* < 0.001) time-dependent difference in mean plasma IFNγ and IL-1β concentrations within piglets from Trial 2020. Plasma IFNγ concentrations were highest at D0 and decreased (*p* < 0.001) by D14 (Figure 3A). The lowest plasma IFNγ concentrations occurred at D63 (*p* < 0.001). In contrast, IL-1β did not show age-dependent changes between D0 and D28, however, D63 concentrations were significantly lower (*p* < 0.001) than D0 (Figure 3B). Temporal variation in plasma cytokines prior to vaccination could limit the use of plasma IFNγ or IL-1β as vaccine response biomarkers if they do not consistently associate with vaccine-induced immunity.

To account for litter-dependent variability in plasma cytokine concentrations, piglets were grouped within litters, and a two-way mixed-effects ANOVA with Tukey's multiple comparisons was conducted using the factors “Litter” and “Time.” There was a significant (*p* < 0.001) interaction between “Litter” and “Time” for mean plasma IFNγ concentration. One litter, “653–3E” (*n* = 11), had consistently higher concentrations of plasma IFNγ when compared to the other 5 litters at D0, D7, D14, D21 (*p* < 0.001), and D28 (*p* < 0.01) (Figure 3C). This difference in plasma IFNγ between Litter 653–3E and the other 5 litters waned and was not significant (*p* < 0.05) at D63. Litter 720–1E (*n* = 12) had higher (*p* < 0.05) plasma IFNγ concentration than 3 other litters at D0, but these differences were not consistently observed at subsequent time-points. Differences in plasma IFNγ concentration among litters were not observed at D63 except for few individual differences that were not consistent with other time-points (Supplementary Table 3). All litters had lower (*p* < 0.05) plasma IFNγ concentration on D63 compared to D0 except Litter 269–2F (*n* = 8), which only showed a trend (*p* = 0.07) toward decreased plasma IFNγ concentrations (Figure 3C).

A two-way mixed-effects ANOVA using the factors “Litter” and “Time” was conducted to identify differences in mean plasma IL-1β concentrations among litters within the second trial. There was a significant (*p* < 0.001) interaction between “Litter” and “Time” for IL-1β concentrations. A comparison among litters revealed Litter 653–3E had significantly higher plasma IL-1β concentrations than the other 5 litters at D0 (*p* < 0.005), 4 litters at D7 and D14 (*p* < 0.005), and 4 litters at D21 and D28 (*p* < 0.05) (Figure 3D). At D63, no litter-dependent differences (*p* > 0.05) were observed among all litters. All litters had lower (*p* < 0.01) plasma IL-1β concentrations at D63 compared to D0, and 4 litters had lower (*p* < 0.05) plasma IL-1β concentrations at D63 compared to all other time-points. Litter 560–2F (*n* = 12) had significantly lower (*p* < 0.005) mean plasma IL-1β concentration than three other litters at D63, but this difference was not apparent at any other time-point (Supplementary Table 4).

Routine monitoring and clinical assessment of piglets throughout Trial 2020 did not identify clinical signs of infection, physical injury, or trauma. Thus, elevated plasma IFNγ and IL-1β, specifically within Litter 653–3E, are likely not due to apparent illness. As we did not perform a complete diagnostic evaluation on these animals it does not preclude the possibility that an asymptomatic infection affecting this specific sow and/or her piglets could be responsible for the elevated pro-inflammatory cytokine levels observed.

Sows had a parity of 0–3 and no relationship between parity and cytokine concentrations was found. Observing a litter (Litter 653–3E) with elevated plasma cytokines compared to other litters with unimpaired vaccine-induced serum IgG titers showed that high cytokine concentrations at the time of vaccination do not always classify piglets as low vaccine responders. However, the elevated IFNγ and IL-1β within this biologically related litter from Trial 2020 may have different roles than the individual LR piglets from Trial 2015. Data from both trials suggests that the plasma cytokines may be situationally dependent biomarkers for predicting vaccine responsiveness.

### Relationship Between Body Weight and Vaccine Responsiveness

**Figure 4:**
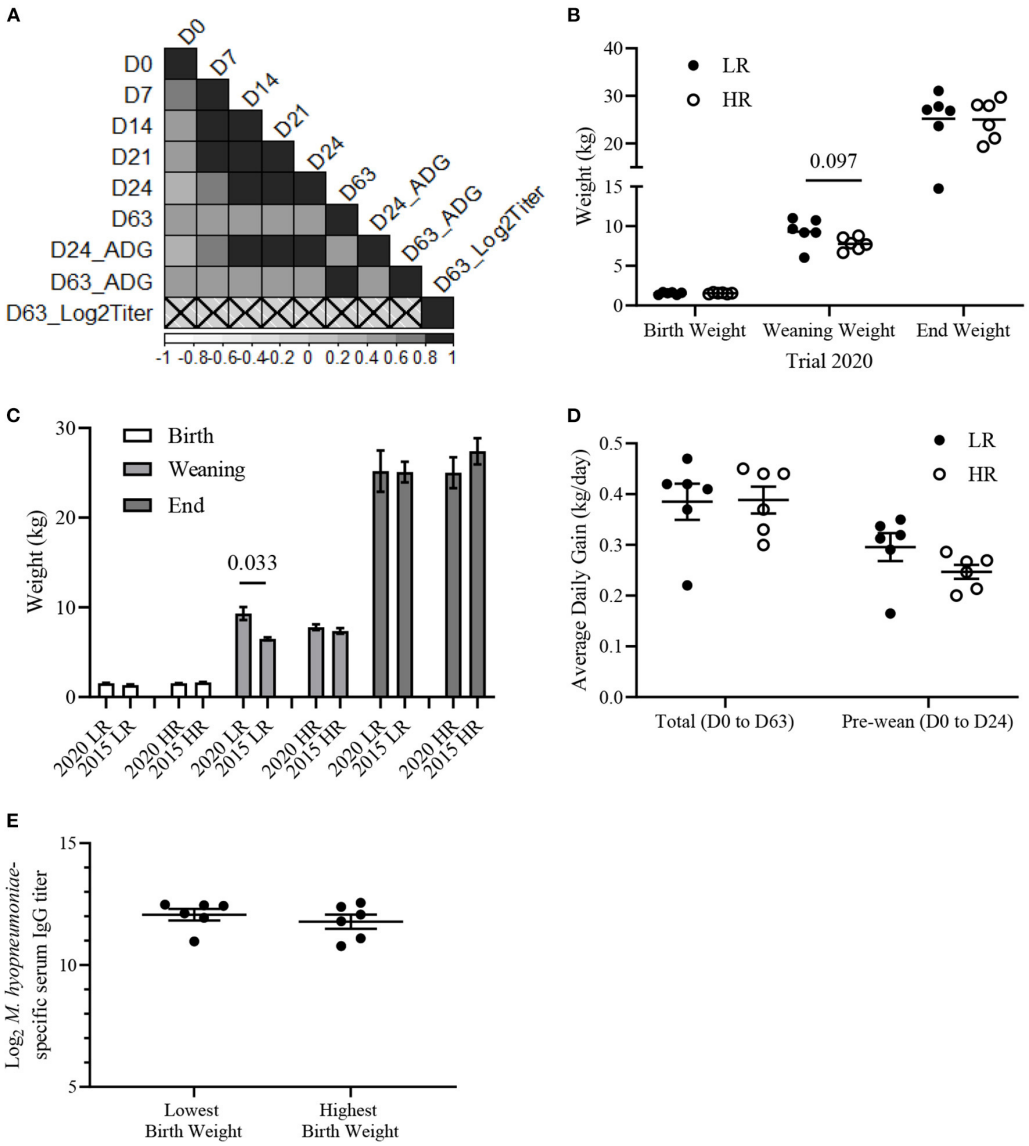
*Mycoplasma hyopneumoniae-*specific serum IgG titers do not associate with body weights of piglets from Trial 2020. **(A)** Pearson correlation matrix of body weights at Day 0 (D0), D7, D14, D21, D24, D63, pre-weaning average daily gain (D24_ADG), total average daily gain (D63_ADG) and Log_2_ *M. hyopneumoniae*-specific serum IgG titer (D63_Log2Titer) from Trial 2020 (*n* = 67). Scale represents Pearson coefficients. Crosses designate insignificant (*p* > 0.05) correlations. **(B)** Mean (±SEM) body weight (kg) of low (LR; *n* = 6; filled circles) and high (HR; *n* = 6; empty circles) at birth (D0), weaning (D24), and 63-days of age (D63). **(C)** Mean (±SEM) body weight (kg) of LR and HR from Trial 2020 and Trial 2015 on D0, D24, and D63. **(D)** Mean (± SEM) average daily gain (kg/day) of LR and HR from Trial 2020 from birth to the end of trial (D0–D60) and from birth to weaning (D0–D24). Average daily gain = [Body weight (kg)—Birth weight (kg)]/age in days (days). **(E)** Mean (±SEM) Log_2_ *M. hyopneumoniae*-specific serum IgG titer of piglets with the lowest (*n* = 6) and highest (*n* = 6) weight at D0. Differences between groups determined using a two-tailed, unpaired Student's *t*-test with Welch's correction.

Body weight is a commonly used metric for evaluating livestock health and likelihood of survival (39). Therefore, we investigated the relationship between piglet growth and vaccine responsiveness. A Pearson correlation analysis was conducted to determine if body weight and average daily gain (ADG) metrics were associated with vaccine-induced antibody responses. Piglets from Trial 2020 showed a positive (*r*^2^ > 0.4) correlation (*p* < 0.05) between body weights at each time-point (D0, D7, D14, D21, D28, and D63) and ADG from birth to weaning (D0–D24) and from birth until the end of the trial (D0–D63) (Figure 4A). There were no significant correlations (*p* > 0.1) between D63 serum IgG titers and body weight or ADG. Previously, we observed LR from Trial 2015 had lower body weight at birth (D0) and weaning (D24) compared to HR, but no difference at 63-days of age (D63) (31). Here, there was no difference in body weight between HR and LR from Trial 2020 at D0 (*p* = 0.91) or D63 (*p* = 0.95) (Figure 4B). While there was a trend of LR having a higher weaning weight than HR (*p* = 0.097), this trend was not consistent with other time points (Figure 4B). To determine if HR or LR differed in growth between Trial 2015 and Trial 2020, we compared body weights of HR and LR between trials. No difference (*p* > 0.05) in body weight was detected between HRs within Trial 2020 and Trial 2015 on D0, D24, and D63 (Figure 4C). However, the LR from Trial 2020 had a higher body weight (*p* < 0.05) at D24 than LR from Trial 2015, but this difference was not consistent at D0 or D63. Additionally, there was no difference (*p* > 0.05) in ADG between HR and LR from Trial 2020 between D0 and D24 or D0 and D63 (Figure 4D). To test further if birth weight was associated with vaccine responsiveness, piglets with the lowest (*n* = 6) and highest (*n* = 6) birth (D0) weights within piglets from Trial 2020 were stratified. Comparative analysis revealed no difference (*p* > 0.05) in D63 serum IgG titers between the highest and lowest birth weight piglets (Figure 4E). Altogether, there were no significant associations between birth weight or weaning weight with vaccine-induced antibody responses in the Trial 2020.

## Discussion

With increasing restrictions on antibiotic use in livestock, there is a greater need for alternative approaches to minimize the occurrence and impact of infectious diseases (40). Vaccination remains one of the most effective methods for reducing infectious disease outbreaks in livestock, however, a potential limitation to the effectiveness of these programs is that variation in individual animal responses to vaccination can result in vaccine unresponsive individuals within a population. These individuals, which can range from poor responses “low responders” to failure to seroconvert “non-responders,” can potentially transmit pathogens and compromise the protection afforded by herd immunity. Effective methods to preemptively identify vaccine unresponsive individuals within a population, prior to vaccination, could mitigate this problem.

We previously reported that vaccine-induced serum IgG titers which were below the threshold of seroconversion were associated with pro-inflammatory cytokine signaling within peripheral blood mononuclear cells, elevated plasma interferon-gamma (IFNγ) concentrations, and low birth weight. These observations suggested that plasma IFNγ concentration and birth weight might serve as pre-vaccination biomarkers of vaccine unresponsiveness. To test this hypothesis, we vaccinated an independent cohort of piglets with the same commercial *M. hyopneumoniae* bacterin (RespiSure-One) to determine if these pre-vaccination markers were consistently associated with post-vaccination antibody responses. Plasma IFNγ and IL-1β concentrations at the time of vaccination did not correlate with vaccine-induced serum IgG titers, and no significant differences in plasma cytokine concentrations, body weight, or piglet growth were found between HR and LR prior to and following vaccination.

When comparing the results of these two investigations, there are several potentially confounding variables to consider. Trial 2015 consisted of piglets (*n* = 117) selected from twenty litters, which is almost double the number of piglets (*n* = 67) selected from only six litters enrolled in Trial 2020. The larger sample size could allow for a wider range of vaccine-induced antibody titers, particularly if vaccine unresponsiveness is an infrequent event. For example, ~5–10% of persons vaccinated against hepatitis B virus are non-responders who fail to mount protective levels of anti-HB titers (41, 42). Recently, a large population (*n* = 278) of piglets born from 47 sows was vaccinated with an inactivated *M. hyopneumoniae* vaccine (Stellamune, Elanco), at 28-days of age and variable *M. hyopneumoniae*-specific IgG titers were observed at 21-, 35-, and 118-days post-vaccination. The results from Blanc et al. aligned with the current study, as the population from that study had variable vaccine-induced antibody responses, yet all piglets were seropositive (S/P > 0.4) 35-days post-vaccination (4). Overall, we find that vaccination with RespiSure-One can result in variable vaccine-induced antibody responses and the level of seroconversion can be either complete, or insufficient at 35-days post vaccination.

Another potential issue is that Trial 2015 and Trial 2020 were conducted at different facilities 5 years apart. Despite efforts to optimize vaccine formulation to ensure a consistent and reproducible immune response within a population, individual-animal differences in vaccine responses can occur (3, 4, 10). In the current study (Trial 2020), there was an 8-fold range in vaccine-induced IgG titers 35-days following vaccination. This distribution of responses had far less variation compared to our previous investigation (Trial 2015), which reported a 64-fold difference between HR and LR (31). Both trials used RespiSure-One containing Amphigen adjuvant (lot numbers not recorded), and there was no evidence of vaccine formulation changes within this timeframe (based on personal communication with the Associate Director of Zoetis, USA). Further discrepancies in the variation of serum IgG titers between Trial 2015 and Trial 2020 could be a consequence of environmental differences between the two facilities that housed these two cohorts, such as variation in animal handling practices that triggered a stress response (43, 44) or differences in diet and microbiome composition. For example, the microbiota composition of weaned piglets can vary among facilities (45) and may impact vaccine-induced antibody responses similar to other studies in humans, mice, and other pig populations (21, 46, 47). While vaccine spoilage or improper injection can result in poor vaccine-induced responses, it is unlikely that this occurred as piglets were vaccinated in multiple batches at different times of the year for both Trial 2015 and Trial 2020, and no differences in serum IgG titers were found among batches.

One of the key distinguishing characteristics between these two trials is that the LR cohort from Trial 2020 may not be phenotypically comparable to the seronegative LR from Trial 2015. The LR from the Trial 2020 (titer range = 10.0–10.4) are considered seropositive for *M. hyopneumoniae*-specific serum antibody responses with a post-vaccination S/P ratio > 0.4 (approximately a Log_2_ endpoint titer of 9.7) based on the ELISA manufacturer's instructions (IDEXX, Inc.). Comparatively, the LR from Trial 2015 (titer range = 5.8–7.7) were classified as seronegative vaccine responders. Thus, the biomarkers discovered with piglets from the first trial might not be applicable to the LR cohort selected in the second trial.

In our previous study, a failure to develop vaccine-induced antibody responses was associated with a lower birth weight (31). Lower birth weight has been associated with decreased humoral responses following vaccination of human infants with hepatitis B virus and tetanus toxin vaccines (35, 36, 48). Within the livestock industry, the selection of hyper-prolific sows to farrow large litter sizes has led to greater intra-litter variation in birth weight and pre-weaning mortality of low-birth-weight piglets (39, 49). As a consequence, low birth weight piglets may be disadvantaged due to competition among littermates, leading to reduced colostrum intake and a greater risk for impaired immune functions (50, 51). While Trial 2015 revealed both birth and weaning weight were significantly associated with vaccine responses, a similar association was not observed in the current investigation. This result was unexpected given that Trial 2015 included only piglets of average birth weights within each litter, whereas Trial 2020 included no such selection criteria (31). This discrepancy in selection criteria did not appear to affect the variability of piglet birth weights as there was no observed difference in piglet birth weight distribution between Trial 2015 and Trial 2020 (data not shown). Therefore, even though the Trial 2020 LR may not accurately represent seronegative vaccine responders, the presence of low-birth-weight piglets with seropositive serum IgG titers implies that piglet birth weight is not a robust predictor of impaired vaccine-induced antibody responses.

We previously detected higher concentrations of plasma IFNγ and IL-1β prior to vaccination within LR than HR piglets in Trial 2015, suggesting these plasma cytokines may be biomarkers of vaccine responsiveness (31). IFNγ plays a crucial role in Th1 responses, activating macrophages, and promoting natural killer cell activity (52). Therefore, we hypothesized that elevated levels of IFNγ and other pro-inflammatory cytokines signified an increased inflammatory state in non-responders prior to vaccination (31). The hypothesis that immune activation prior to vaccination can influence the magnitude of vaccine responses has been presented previously. In humans, greater frequencies of activated natural killer cells and pro-inflammatory monocytes were present in the blood prior to vaccination of an African cohort with impaired cellular and neutralizing antibody titers responses following yellow fever 17D virus vaccination when compared to a Swiss cohort with unimpaired vaccine responses (34). In addition, high serum levels of the pro-inflammatory cytokine, tumor necrosis factor α, has been negatively associated with influenza-specific haemagglutination inhibition titers in older humans following influenza virus vaccination (19). Similarly, activation of innate immune cells and the upregulation of both pro-inflammatory gene signaling pathways and pro-inflammatory cytokines were associated with reduced seroconversion to a hepatitis B vaccine in older humans (24).

While the above studies provide evidence that pre-vaccination inflammatory processes can negatively impact vaccine-induced antibody responses, plasma cytokine analyses in Trial 2020 revealed that plasma cytokine concentrations did not correlate with vaccine-induced antibody responses. Indeed, pre-vaccination serum cytokine levels in low vaccine responders has not always correlated with vaccine-induced immune responses (24, 53). As well, other studies associating inflammation and vaccine-induced responses were conducted in human and mouse models, and may not translate to young, developing piglets. However, it is possible that plasma IFNγ and IL-1β detected within LR may have had different biological roles between Trial 2015 and Trial 2020. Elevated pro-inflammatory cytokines can reflect both harmful (tissue damage, autoimmunities, necrosis) and beneficial (increased microbial killing, response to pathogenic stimuli) inflammatory mechanisms (52, 54). Whether Trial 2015 LR IFNγ concentrations indicate an underlying innate-immune activation, while Trial 2020 LR IFNγ concentrations indicate homeostatic inflammation is unknown; future investigations into this relationship should include baseline samples at multiple time points prior to vaccination. Ultimately, as Trial 2020 lacked a cohort of non-responders comparable to those in Trial 2015 LR, the utility of IFNγ as a biomarker for antibody responsiveness may be limited to the extremes of the vaccine-induced antibody response phenotype.

While plasma cytokines did not explain variability in vaccine-induced antibody responses, the current study revealed temporal changes in plasma IFNγ and IL-1β concentrations during the first 63 days of age. Plasma IFNγ and IL-1β concentrations were observed to decrease over the first 4 weeks of life for all piglets in Trial 2020, and concentrations were lowest at 2 months. Decreasing levels of IFNγ within the entire cohort suggests a consistent biological process within all litters. A previous investigation by Nguyen et al. also reported that plasma cytokines, such as IFNγ, IL-6, and IL-4, decrease in piglets during the first 2 weeks following birth. However, these decreases in plasma cytokine concentrations were not observed for all cytokines (55). In addition, the pro-inflammatory cytokines IL-1β and IL-6, but not TNFα, were elevated in the serum of human infants at 1-day of age and declined significantly from 1- to 40-days of age (56). Though elevated plasma cytokines could reflect an endogenous immune response following birth, they may originate from the exogenous absorption of maternal cytokines in colostrum prior to gut closure, which was proposed to aid in immune development for naïve bovine and human neonates (55, 57, 58). Maternal transfer of cytokines from sow to piglet has been observed elsewhere and may be involved in protection against pathogens by inducing mucosal immune responses and endogenous cytokine production within the neonate (59). Colostral cytokines were not analyzed in this study, and therefore, the origin of the plasma cytokines within piglets remains inconclusive. Thus, the extent to which IFNγ and IL-1β impact vaccine responsiveness may be situationally dependent on other undetermined variables; piglets with elevated IFNγ concentrations may have a propensity for impaired vaccine-induced antibody responses, yet elevated IFNγ concentrations do not necessarily result in or predict impaired vaccine-induced antibody responses.

The current study also found significant litter-dependent effects on plasma cytokine concentrations that were most pronounced shortly after birth. The occurrence of one litter (653–3E) with much higher plasma IFNγ and IL-1β offered an opportunity to test the hypothesis that a pro-inflammatory environment impairs vaccine responsiveness. Litter-specific differences have been detected for other immune parameters such as IFNγ/IL-10 production following *ex vivo* stimulation of porcine PBMCs (60). The serum IgG titers within Litter 653–3E were comparable to the other litters, indicating no significant consequences of elevated IFNγ on vaccine-induced antibody responses. High IFNγ levels of this magnitude were observed in all piglets of the litter, possibly reflecting a genetic or epigenetic characteristic of the litter or an immunomodulatory challenge to the sow prior to/during farrowing such as stress (44). Both plasma IFNγ and IL-1β levels in Litter 653–3E returned to levels comparable to the other litters by 63 days of age, suggesting that the elevated cytokines reflect a prolonged, but ultimately resolved, response to an early event. While temporal variations in plasma cytokines may reflect developmental or immune processes within the piglet that require additional investigation, effective plasma cytokines biomarkers of vaccine responsiveness need to be predictive independent of temporal biological changes.

The main limitation of the current study was an absence of seronegative vaccine responders that could possibly validate plasma cytokines and birth weight as predictive biomarkers of vaccine responsiveness (31). Therefore, future studies exploring immune correlates of vaccine responsiveness may need a larger population size to increase the probability of encountering seronegative vaccine responders. The current study also utilized vaccine-specific serum IgG titers at 35 days post-vaccination as a metric of vaccine responsiveness. This was to align with our previous study, but future trials could incorporate cell-mediated immune responses as metrics of vaccine responsiveness. In addition, future studies should collect immune tissue for functional analysis needed to supplement findings related to changes in plasma cytokine concentrations. For example, collecting blood leukocytes from piglets after birth may be used for gene expression analysis of interferon-stimulated genes to confirm that circulating plasma cytokines in piglets have functional consequences. Finally, longitudinal analysis of subsequent litters from the sows used in this study may have revealed whether genetic/maternal effects or environmental effects drove litter-specific differences in plasma cytokines.

Identifying vaccine unresponsive individuals may permit additional biosecurity measures, such as isolation from the rest of the herd or implementation of other disease management regimens to enhance disease protection. While comparing the results between our two vaccination trials reveals inconsistent relationships between vaccine responsiveness, plasma cytokines, and piglet body weight, independent studies are necessary to evaluate the robustness of these putative biomarkers in outbred animal models. In addition, further efforts are required to understand the specific situations and mechanisms by which temporal elevations in circulating cytokines influence adaptive immunity.

## Data Availability Statement

The original contributions presented in the study are included in the article/Supplementary Material, further inquiries can be directed to the corresponding author/s.

## Ethics Statement

The animal study was reviewed and approved by the experimental protocol (AUP20190084) was approved by the University of Saskatchewan Animal Care and Use Committee in accordance with the Canadian Council on Animal Care guidelines.

## Author Contributions

SL: conceptualization, validation, formal analysis, investigation, writing—original draft, writing—review and editing, and visualization. AF and PG: conceptualization and writing—review and editing. ES: investigation and project administration. SN: conceptualization, resources, writing—original draft, writing—review and editing supervision, and funding acquisition. All authors contributed to the article and approved the submitted version.

## Funding

This research was enabled by individual Discovery Grants from the Natural Sciences and Engineering Research Council of Canada (NSERC) to SN (RGPIN 05690-2018). VIDO receives operational funding from the Government of Saskatchewan through Innovation Saskatchewan and the Ministry of Agriculture and from the Canada Foundation for Innovation through the Major Science Initiatives for its CL3 facility (InterVac).

## Conflict of Interest

The authors declare that the research was conducted in the absence of any commercial or financial relationships that could be construed as a potential conflict of interest.

## Publisher's Note

All claims expressed in this article are solely those of the authors and do not necessarily represent those of their affiliated organizations, or those of the publisher, the editors and the reviewers. Any product that may be evaluated in this article, or claim that may be made by its manufacturer, is not guaranteed or endorsed by the publisher.
