# Supplementary material for: Plasma Cytokines and Birth Weight as Biomarkers of Vaccine-Induced Humoral Responses in Piglets
Source: Front Vet Sci. 2022 Jul 13;9:922992. doi: 10.3389/fvets.2022.922992 (PMC9325413; doi:10.3389/fvets.2022.922992)
Supplement: Supplementary file 1 [file Table_1.docx]

**Supplementary Tables**

|  |
| --- |

**Supplementary Table 1: Interferon-gamma (IFN**γ**) concentrations (pg/mL) of individual animals for each Day. Out of range (OOR) values were not included and treated as "zero".**

| **Interferon gamma (pg/mL)** | | | | | | | |
| --- | --- | --- | --- | --- | --- | --- | --- |
| **Pig ID** | **Litter ID** | **Day 0** | **Day 7** | **Day 14** | **Day 21** | **Day 28** | **Day 63** |
| Y1 | 423-3E | 123.62 | 101.91 | 38.1 | OOR < | OOR < | OOR < |
| Y2 | 423-3E | 391.99 | 153.86 | 138.95 | 157.49 | 100.9 | 42.12 |
| Y3 | 423-3E | 176.77 | 196.84 | 100.5 | 90.18 | 118.54 | OOR < |
| Y4 | 423-3E | 352.285 | 138.83 | 228.08 | 200.25 | 138.57 | 69.94 |
| Y5 | 423-3E | 223.82 | 291.025 | 105.1 | 154.16 | 113.71 | 45.3 |
| Y6 | 423-3E | 395.35 | 246.185 | 72.13 | 140.32 | 123.53 | 47.51 |
| Y7 | 423-3E | 405.185 | 188.87 | 138.5 | 172.59 | 186.12 | 44.895 |
| Y8 | 423-3E | 196.08 | 137.12 | 84.305 | 208.615 | 115.12 | OOR |
| Y9 | 423-3E | 175.23 | 208 | 188.75 | 155.11 | 98.39 | OOR < |
| Y10 | 423-3E | 267.85 | 203.62 | 895.225 | 251.44 | 153.99 | OOR < |
| Y11 | 423-3E | 209.535 | 184.73 | 219.37 | 184.89 | 169.81 | 34.2 |
| Y12 | 423-3E | 117.945 | 212.78 | 139.67 | 115.91 | 106.68 | 29.3 |
| Y13 | 423-3E | 199.935 | OOR < | 228.46 | 95.55 | 182.13 | 31.7 |
| Y14 | 423-3E | 293.23 | 214.81 | 144.17 | 167.95 | 130.62 | OOR < |
| Y15 | 560-2F | 310.75 | 253.34 | 103.33 | 179.69 | 142.76 | 30.365 |
| Y16 | 560-2F | 252.97 | 348.24 | 188.75 | 209.18 | 196.87 | 67.72 |
| Y17 | 560-2F | 213.875 | 720.48 | 164.93 | 157.68 | 164.43 | OOR < |
| Y18 | 560-2F | 300.14 | 121.66 | 133.92 | 134 | 148.33 | OOR < |
| Y19 | 560-2F | 252.52 | 514.78 | 88.13 | 91.06 | 71.21 | OOR < |
| Y20 | 560-2F | 260.58 | 197.38 | 167.21 | 198.09 | 178.41 | 32.3 |
| Y21 | 560-2F | 440.24 | 690.06 | 218.34 | 151.59 | 111.6 | OOR < |
| Y22 | 560-2F | 408.005 | 137.65 | 105.63 | 195.08 | 128.52 | 28.895 |
| Y24 | 560-2F | 244.625 | 97.92 | 263.08 | 137.35 | 147.55 | OOR < |
| Y25 | 560-2F | 266.97 | 99.28 | 135.71 | 142.23 | 114.06 | OOR < |
| Y26 | 560-2F | 233.2 | 335.48 | 170.13 | 153.88 | 121.69 | OOR < |
| Y27 | 560-2F | 224.79 | 349.82 | 229.49 | 231.1 | 160.69 | OOR < |
| Y41 | 653-3E | 1779.61 | 2347.43 | 1702.35 | 1125.32 | 866.34 | 70.19 |
| Y42 | 653-3E | 1498.77 | 1511.79 | 1008.37 | 638.32 | 404.05 | 29.26 |
| Y43 | 653-3E | 2892.495 | 1758.21 | 1069.95 | 708.8 | 483.54 | 38.28 |
| Y44 | 653-3E | 2131.585 | 1444.73 | 1127.16 | 836.25 | 551.51 | 52.9 |
| Y45 | 653-3E | 2232.045 | 1465.4 | 1111.4 | 1121.29 | 846.48 | 104.77 |
| Y46 | 653-3E | 2204.91 | 1376.765 | 1010.55 | 839.79 | 558 | 49.69 |
| Y47 | 653-3E | 2351.105 | 1489.715 | 1005.93 | 845.89 | 502.94 | 55.91 |
| Y48 | 653-3E | 2704.08 | 1830.1 | 1027.01 | 941.81 | 721.36 | 88.75 |
| Y50 | 653-3E | 1799.87 | 1206.8 | 842.53 | 608.975 | 226.53 | OOR < |
| Y51 | 653-3E | 1073.82 | 1317.81 | 615.37 | 398.49 | 113.08 | 28.14 |
| Y52 | 653-3E | 2308.27 | 1713.555 | 1457.32 | 950.65 | 837.05 | 87.73 |
| P1 | 269-2F | OOR < | 118.94 | 89.91 | 90.61 | 195.44 | 38.225 |
| P4 | 269-2F | OOR < | 146.7 | 124.09 | 146.24 | 113.93 | 28.2 |
| P5 | 269-2F | 275.155 | 117.185 | 150.9 | 98.93 | 60.725 | 26.005 |
| P6 | 269-2F | 126.54 | 283.12 | 70.99 | 61.22 | 61.91 | 30.23 |
| P7 | 269-2F | 168.98 | 276.16 | 90.77 | 87.22 | 50.39 | 35.43 |
| P10 | 269-2F | 133 | 309.29 | 80.93 | 66.91 | 53.29 | OOR < |
| P11 | 269-2F | 118.53 | 159.76 | 76.76 | 46.06 | 58.305 | 31.86 |
| P12 | 269-2F | 99.01 | 308.59 | 117.97 | 217.42 | 91.93 | 40.01 |
| P26 | 720-1E | OOR < | 467.99 | 290.26 | 208.42 | 244.19 | 63.05 |
| P27 | 720-1E | 575.49 | 263.075 | 165.67 | 127.58 | 154.97 | OOR < |
| P28 | 720-1E | 323.23 | 385.37 | 178.1 | 122.88 | 148.39 | OOR |
| P29 | 720-1E | 382.93 | 411.37 | 157.38 | 131.765 | 125.64 | OOR < |
| P31 | 720-1E | 620.235 | 473.56 | 166.92 | 129.83 | 225.56 | 58.255 |
| P32 | 720-1E | 449.285 | 430.63 | 185.6 | 158.24 | 192.33 | 41.44 |
| P33 | 720-1E | 304.82 | 332.06 | 152.5 | 134.11 | 130.505 | OOR < |
| P34 | 720-1E | 580.23 | 260.35 | 169.67 | 143.79 | 196.85 | 56.42 |
| P36 | 720-1E | 382.485 | 313.94 | 123.48 | 114.5 | 128.24 | 36.24 |
| P37 | 720-1E | 442.11 | 384.205 | 257.17 | 222.26 | 194.28 | 68.55 |
| P38 | 720-1E | 483.705 | 376.65 | 260.185 | OOR < | 159.585 | 48.16 |
| P39 | 720-1E | 541.37 | 349.675 | 317.01 | OOR < | 289.385 | 74.88 |
| P40 | 440-1E | 488.52 | 308.25 | 100.26 | 87.57 | 95.205 | OOR < |
| P42 | 440-1E | 321.5 | 291.785 | 193.86 | 93.24 | 126.39 | 24.605 |
| P43 | 440-1E | 331.345 | 941.55 | 145.16 | 104.72 | 107.135 | OOR < |
| P44 | 440-1E | 409.5 | 318.865 | 118.46 | 92.16 | 108.125 | OOR < |
| P46 | 440-1E | 365.7 | 279.665 | 113.3 | 52.6 | 41.37 | OOR < |
| P47 | 440-1E | 367.87 | 208.78 | 113.49 | 96.94 | 71.78 | OOR < |
| P49 | 440-1E | 315 | 224.83 | 343.375 | 109.84 | 110.51 | 27.24 |
| P50 | 440-1E | 259.41 | 245.55 | 321.725 | 86.23 | 87.735 | 32 |
| P51 | 440-1E | 257.23 | 226.325 | 204.91 | 88.895 | 59.47 | OOR < |
| P52 | 440-1E | 222.105 | 206.46 | 72.975 | 116.43 | 87.45 | OOR < |

**Supplementary Table 2: Interleukin 1-beta (IL-1**β**) concentrations (pg/mL) of individual animals for each Day. Out of range (OOR) values were not included and treated as "zero".**

| **Interleukin 1-beta (pg/mL)** | | | | | | | |
| --- | --- | --- | --- | --- | --- | --- | --- |
| **Litter ID** | **Pig ID** | **Day 0** | **Day 7** | **Day 14** | **Day 21** | **Day 28** | **Day 63** |
| 423-3E | Y1 | 1085.64 | 1016.57 | 673.01 | 460.92 | 358.98 | OOR < |
| 423-3E | Y2 | 717.56 | 492.98 | 691.1 | 674.62 | 439.58 | 301.39 |
| 423-3E | Y3 | 545.72 | 764.71 | 603.9 | 613.14 | 631.21 | 246.78 |
| 423-3E | Y4 | 728.015 | 446.27 | 626.34 | 612.52 | 684.47 | 306.04 |
| 423-3E | Y5 | 562.87 | 898.15 | 612.88 | 661.83 | 716.32 | 338.51 |
| 423-3E | Y6 | 730.41 | 749.095 | 467.56 | 694.3 | 673.48 | 328.77 |
| 423-3E | Y7 | 916.89 | 479.07 | 748.1 | 726.07 | 816.28 | 307.18 |
| 423-3E | Y8 | 499.75 | 588.43 | 705.72 | 330.485 | 726.65 | 270.19 |
| 423-3E | Y9 | 577.655 | 737.68 | 860.815 | 525.12 | 670.18 | 272.56 |
| 423-3E | Y10 | 397.83 | 733.42 | 1066.89 | 896.79 | 711.18 | 206.8 |
| 423-3E | Y11 | 468.14 | 422 | 1038.92 | 769.15 | 1016.56 | 410.96 |
| 423-3E | Y12 | 404.285 | 1041.91 | 950.79 | 725.38 | 898.18 | 356.63 |
| 423-3E | Y13 | 537.37 | OOR < | 951.03 | 376.35 | 772.91 | 306.77 |
| 423-3E | Y14 | 815.5 | 705.88 | 697.26 | 917.32 | 891.69 | 251.76 |
| 560-2F | Y15 | 828.74 | 1174.49 | 736.03 | 1109.98 | 929.97 | 187.42 |
| 560-2F | Y16 | 809.17 | 1409.75 | 1060.05 | 992.76 | 1018.67 | 185.125 |
| 560-2F | Y17 | 758.75 | 1690.26 | 1091.93 | 1088.82 | 944.82 | 133.62 |
| 560-2F | Y18 | 720.54 | 684.71 | 858.57 | 862.155 | 950.07 | 165.94 |
| 560-2F | Y19 | 930.88 | 1791.7 | 787.62 | 821.73 | 674.16 | 59.245 |
| 560-2F | Y20 | 692.24 | 958.78 | 1006.5 | 1209.46 | 1065.61 | 170.29 |
| 560-2F | Y21 | 1197.305 | 2698.8 | 1293.24 | 1090.05 | 861.14 | 134.01 |
| 560-2F | Y22 | 632.55 | 813.57 | 757.6 | 1157.44 | 888.11 | 312.155 |
| 560-2F | Y24 | 639.06 | 557.515 | 1312.61 | 1027.06 | 957.45 | 153.2 |
| 560-2F | Y25 | 913.38 | 484.985 | 930.85 | 1023.46 | 864.45 | 125.94 |
| 560-2F | Y26 | 757.06 | 1157.55 | 1065.93 | 1059.99 | 851.99 | 132.29 |
| 560-2F | Y27 | 610.3 | 1282.96 | 1239.43 | 1279.63 | 961.34 | 213.05 |
| 653-3E | Y41 | 1812.11 | 2175.68 | 2087.24 | 1774.11 | 1367.04 | 303.73 |
| 653-3E | Y42 | 1713.32 | 1633.75 | 1605.14 | 1320.7 | 977.44 | 81.12 |
| 653-3E | Y43 | 2647.265 | 1806.28 | 1810.22 | 1019.27 | 1048.54 | 223.28 |
| 653-3E | Y44 | 2238.88 | 1836.45 | 1901.33 | 1114 | 1185.82 | 304.96 |
| 653-3E | Y45 | 2344.805 | 1763.42 | 1860.93 | 1398.52 | 1404.03 | 456.38 |
| 653-3E | Y46 | 2147.21 | 1771.345 | 1744.42 | 1208.2 | 1138.05 | 309.16 |
| 653-3E | Y47 | 2310.12 | 1767.08 | 1744.49 | 1111.03 | 1132.74 | 336.59 |
| 653-3E | Y48 | 2442.62 | 1980.27 | 1840.09 | 1200.7 | 1265.52 | 414.1 |
| 653-3E | Y50 | 1828.465 | 1622.86 | 1637.07 | 827.405 | 719.28 | 128.7 |
| 653-3E | Y51 | 937.23 | 1478.45 | 1414.73 | 796.44 | 470.52 | 110.72 |
| 653-3E | Y52 | 2206.87 | 1808.12 | 2017.38 | 1263.26 | 1395.34 | 421.77 |
| 269-2F | P1 | OOR < | 750.46 | 916.53 | 848.165 | 579.66 | 549.495 |
| 269-2F | P4 | OOR < | 652.96 | 1249.86 | 968.105 | 766.76 | 385.66 |
| 269-2F | P5 | 555.52 | 810 | 1318.76 | 840.85 | 548.55 | 309.3 |
| 269-2F | P6 | 654.49 | 1135.39 | 733.845 | 603.42 | 639.08 | 361.41 |
| 269-2F | P7 | 611.39 | 1165.77 | 956.09 | 801.79 | 386.66 | 411.45 |
| 269-2F | P10 | 758.43 | 1355.88 | 903.74 | 839.275 | 362.2 | 263.86 |
| 269-2F | P11 | 814.145 | 897.93 | 845.97 | 701.11 | 640.58 | 420.92 |
| 269-2F | P12 | 883.255 | 1288.95 | 1205.68 | 1022.23 | 903.98 | 437.33 |
| 720-1E | P26 | 746.975 | 838.35 | 1110.85 | 758.42 | 934.04 | 482.27 |
| 720-1E | P27 | 795.56 | 528.57 | 805.87 | 636.2 | 803.51 | 258.24 |
| 720-1E | P28 | 776.89 | 540.45 | 880.75 | 698.07 | 823.31 | 298.26 |
| 720-1E | P29 | 719.765 | 585.01 | 784.3 | 688.27 | 771.09 | 175.31 |
| 720-1E | P31 | OOR < | 629.32 | 764.46 | 599.31 | 799.11 | 540.405 |
| 720-1E | P32 | 829.58 | 723.48 | 822.375 | 826.86 | 748.905 | 407.66 |
| 720-1E | P33 | 612.07 | 659.12 | 780.62 | 670.64 | 665.72 | 265.18 |
| 720-1E | P34 | 761.45 | 495.19 | 466.38 | 549.46 | 752.925 | 405.86 |
| 720-1E | P36 | 694.1 | 431.97 | 561.3 | 550.97 | 396.14 | 396.14 |
| 720-1E | P37 | 686.15 | 527.86 | 849.55 | 804.71 | 477.07 | 477.07 |
| 720-1E | P38 | 725.96 | 710.57 | 856.905 | OOR < | 760.37 | 415.57 |
| 720-1E | P39 | 798.01 | 743.39 | 527.71 | OOR < | 1039.025 | 399.715 |
| 440-1E | P40 | 1437.74 | 1105.32 | 673.55 | 685.08 | 853.475 | 181.725 |
| 440-1E | P42 | 800.8 | 978.42 | 861.7 | 554.25 | 778.195 | 443.59 |
| 440-1E | P43 | 1163.95 | 1125.09 | 714.48 | 520.42 | 527.65 | 324.97 |
| 440-1E | P44 | 1099.06 | 1024.7 | 751.17 | 573.995 | 783.95 | 304.08 |
| 440-1E | P46 | 1480.87 | 1259.32 | 1171.23 | 598.685 | 458.84 | 81.27 |
| 440-1E | P47 | 1302.56 | 806.79 | 585.985 | 654.025 | 715.08 | 212.79 |
| 440-1E | P49 | 909.98 | 876.71 | 3489.75 | 635.75 | 718.41 | 388.085 |
| 440-1E | P50 | 838.59 | 940.12 | 3200.22 | 636.245 | 596.56 | 189.28 |
| 440-1E | P51 | 725.555 | 825.6 | 780.29 | 654.38 | 608.43 | 399.03 |
| 440-1E | P52 | 796.39 | 800.55 | 571.07 | 708.325 | 761.2 | 301.48 |

**Supplementary Table 3: ANOVA table of Tukey’s Multiple Comparisons for interferon-gamma (IFNγ) concentrations between litters (n=6) within Trial 2020 from D0 to D63.** ^ns^p>0.05, *p<0.05, **p<0.01, ***p<0.005, ****p<0.001.

| **Tukey’s multiple comparisons test** | **Mean difference** | **95% CI of diff.** | **Summary** | **Adjusted P-value** |
| --- | --- | --- | --- | --- |
| D0 | | | | |
| 423-3E vs. 560-2F | -32 | -136.6 to 72.64 | ns | 0.9295 |
| 423-3E vs. 653-3E | -1837 | -2386 to -1287 | **** | <0.0001 |
| 423-3E vs. 269-2F | 98.52 | -23.03 to 220.1 | ns | 0.1482 |
| 423-3E vs. 720-1E | -210.3 | -341.9 to -78.67 | *** | 0.0008 |
| 423-3E vs. 440-1E | -81.76 | -195.9 to 32.39 | ns | 0.2631 |
| 560-2F vs. 653-3E | -1805 | -2353 to -1256 | **** | <0.0001 |
| 560-2F vs. 269-2F | 130.5 | 17.59 to 243.5 | * | 0.0212 |
| 560-2F vs. 720-1E | -178.3 | -301.1 to -55.48 | ** | 0.0026 |
| 560-2F vs. 440-1E | -49.76 | -152.5 to 52.95 | ns | 0.6472 |
| 653-3E vs. 269-2F | 1935 | 1386 to 2485 | **** | <0.0001 |
| 653-3E vs. 720-1E | 1626 | 1076 to 2177 | **** | <0.0001 |
| 653-3E vs. 440-1E | 1755 | 1206 to 2304 | **** | <0.0001 |
| 269-2F vs. 720-1E | -308.8 | -444.3 to -173.3 | **** | <0.0001 |
| 269-2F vs. 440-1E | -180.3 | -300.7 to -59.85 | ** | 0.0029 |
| 720-1E vs. 440-1E | 128.5 | -1.418 to 258.5 | ns | 0.0535 |
| D7 | | | | |
| 423-3E vs. 560-2F | -131.5 | -348.6 to 85.62 | ns | 0.3792 |
| 423-3E vs. 653-3E | -1397 | -1728 to -1066 | **** | <0.0001 |
| 423-3E vs. 269-2F | -24.31 | -141.2 to 92.62 | ns | 0.9742 |
| 423-3E vs. 720-1E | -180.1 | -257.4 to -102.7 | **** | <0.0001 |
| 423-3E vs. 440-1E | -134.5 | -382.8 to 113.7 | ns | 0.458 |
| 560-2F vs. 653-3E | -1265 | -1629 to -901.7 | **** | <0.0001 |
| 560-2F vs. 269-2F | 107.2 | -120.1 to 334.5 | ns | 0.6538 |
| 560-2F vs. 720-1E | -48.57 | -268.1 to 171.0 | ns | 0.9743 |
| 560-2F vs. 440-1E | -3.032 | -299.9 to 293.9 | ns | >0.9999 |
| 653-3E vs. 269-2F | 1373 | 1037 to 1708 | **** | <0.0001 |
| 653-3E vs. 720-1E | 1217 | 884.7 to 1549 | **** | <0.0001 |
| 653-3E vs. 440-1E | 1262 | 887.4 to 1637 | **** | <0.0001 |
| 269-2F vs. 720-1E | -155.8 | -277.8 to -33.74 | ** | 0.01 |
| 269-2F vs. 440-1E | -110.2 | -365.2 to 144.7 | ns | 0.7001 |
| 720-1E vs. 440-1E | 45.53 | -204.1 to 295.2 | ns | 0.9863 |
| D14 | | | | |
| 423-3E vs. 560-2F | 30.33 | -159.1 to 219.8 | ns | 0.9945 |
| 423-3E vs. 653-3E | -894.5 | -1223 to -566.4 | **** | <0.0001 |
| 423-3E vs. 269-2F | 94.09 | -93.35 to 281.5 | ns | 0.5824 |
| 423-3E vs. 720-1E | -7.616 | -198.2 to 183.0 | ns | >0.9999 |
| 423-3E vs. 440-1E | 21.63 | -178.8 to 222.1 | ns | 0.9993 |
| 560-2F vs. 653-3E | -924.8 | -1226 to -623.4 | **** | <0.0001 |
| 560-2F vs. 269-2F | 63.76 | 4.746 to 122.8 | * | 0.0301 |
| 560-2F vs. 720-1E | -37.94 | -112.2 to 36.32 | ns | 0.6107 |
| 560-2F vs. 440-1E | -8.697 | -118.6 to 101.2 | ns | 0.9998 |
| 653-3E vs. 269-2F | 988.6 | 687.8 to 1289 | **** | <0.0001 |
| 653-3E vs. 720-1E | 886.9 | 585.0 to 1189 | **** | <0.0001 |
| 653-3E vs. 440-1E | 916.2 | 610.4 to 1222 | **** | <0.0001 |
| 269-2F vs. 720-1E | -101.7 | -167.5 to -35.92 | ** | 0.0015 |
| 269-2F vs. 440-1E | -72.46 | -178.9 to 33.98 | ns | 0.2623 |
| 720-1E vs. 440-1E | 29.24 | -83.00 to 141.5 | ns | 0.9538 |
| D21 | | | | |
| 423-3E vs. 560-2F | -3.966 | -56.23 to 48.30 | ns | 0.9999 |
| 423-3E vs. 653-3E | -658.5 | -890.0 to -427.0 | **** | <0.0001 |
| 423-3E vs. 269-2F | 59.29 | -18.43 to 137.0 | ns | 0.1833 |
| 423-3E vs. 720-1E | 11.77 | -41.85 to 65.40 | ns | 0.9814 |
| 423-3E vs. 440-1E | 68.25 | 24.18 to 112.3 | ** | 0.0015 |
| 560-2F vs. 653-3E | -654.5 | -885.8 to -423.2 | **** | <0.0001 |
| 560-2F vs. 269-2F | 63.25 | -13.33 to 139.8 | ns | 0.1288 |
| 560-2F vs. 720-1E | 15.74 | -35.20 to 66.68 | ns | 0.9211 |
| 560-2F vs. 440-1E | 72.22 | 31.92 to 112.5 | *** | 0.0004 |
| 653-3E vs. 269-2F | 717.8 | 484.2 to 951.3 | **** | <0.0001 |
| 653-3E vs. 720-1E | 670.3 | 438.9 to 901.7 | **** | <0.0001 |
| 653-3E vs. 440-1E | 726.7 | 496.0 to 957.5 | **** | <0.0001 |
| 269-2F vs. 720-1E | -47.51 | -124.7 to 29.65 | ns | 0.3596 |
| 269-2F vs. 440-1E | 8.964 | -65.42 to 83.35 | ns | 0.9971 |
| 720-1E vs. 440-1E | 56.48 | 13.65 to 99.30 | ** | 0.0079 |
| D28 | | | | |
| 423-3E vs. 560-2F | -6.809 | -46.84 to 33.22 | ns | 0.9943 |
| 423-3E vs. 653-3E | -421.8 | -683.5 to -160.1 | ** | 0.0022 |
| 423-3E vs. 269-2F | 47.96 | -19.03 to 114.9 | ns | 0.2171 |
| 423-3E vs. 720-1E | -48.79 | -103.0 to 5.378 | ns | 0.0916 |
| 423-3E vs. 440-1E | 44.18 | 7.467 to 80.90 | * | 0.0127 |
| 560-2F vs. 653-3E | -415 | -676.9 to -153.2 | ** | 0.0024 |
| 560-2F vs. 269-2F | 54.77 | -13.13 to 122.7 | ns | 0.1419 |
| 560-2F vs. 720-1E | -41.98 | -97.93 to 13.96 | ns | 0.2154 |
| 560-2F vs. 440-1E | 50.99 | 10.96 to 91.03 | ** | 0.0079 |
| 653-3E vs. 269-2F | 469.8 | 206.6 to 733.0 | *** | 0.0008 |
| 653-3E vs. 720-1E | 373 | 110.5 to 635.6 | ** | 0.0053 |
| 653-3E vs. 440-1E | 466 | 204.3 to 727.7 | *** | 0.001 |
| 269-2F vs. 720-1E | -96.75 | -170.8 to -22.71 | ** | 0.0074 |
| 269-2F vs. 440-1E | -3.777 | -70.79 to 63.23 | ns | >0.9999 |
| 720-1E vs. 440-1E | 92.98 | 38.86 to 147.1 | *** | 0.0005 |
| D63 | | | | |
| 423-3E vs. 560-2F | 3.301 | -43.17 to 49.77 | ns | 0.9992 |
| 423-3E vs. 653-3E | -17.44 | -48.81 to 13.92 | ns | 0.4785 |
| 423-3E vs. 269-2F | 10.27 | -7.066 to 27.60 | ns | 0.3703 |
| 423-3E vs. 720-1E | -12.75 | -34.15 to 8.638 | ns | 0.4115 |
| 423-3E vs. 440-1E | 15.17 | -2.687 to 33.03 | ns | 0.107 |
| 560-2F vs. 653-3E | -20.74 | -66.47 to 24.99 | ns | 0.5901 |
| 560-2F vs. 269-2F | 6.969 | -44.02 to 57.96 | ns | 0.9648 |
| 560-2F vs. 720-1E | -16.05 | -62.32 to 30.21 | ns | 0.663 |
| 560-2F vs. 440-1E | 11.87 | -38.86 to 62.61 | ns | 0.8019 |
| 653-3E vs. 269-2F | 27.71 | -2.170 to 57.59 | ns | 0.0733 |
| 653-3E vs. 720-1E | 4.688 | -26.85 to 36.23 | ns | 0.9958 |
| 653-3E vs. 440-1E | 32.61 | 2.610 to 62.62 | * | 0.0316 |
| 269-2F vs. 720-1E | -23.02 | -40.95 to -5.097 | * | 0.0118 |
| 269-2F vs. 440-1E | 4.903 | -7.163 to 16.97 | ns | 0.591 |
| 720-1E vs. 440-1E | 27.93 | 9.520 to 46.33 | ** | 0.0042 |

**Supplementary Table 4: ANOVA table of Tukey’s Multiple Comparisons for interleukin 1-beta (IL-1β) concentrations between litters (n=6) within Trial 2020 from D0 to D63. ^ns^p>0.05, *p<0.05, **p<0.01, ***p<0.005, ****p<0.001.**

| **Tukey’s multiple comparisons test** | **Mean difference** | **95% CI of diff.** | **Summary** | **Adjusted P-value** |
| --- | --- | --- | --- | --- |
| D0 | | | | |
| 423-3E vs. 560-2F | -148.9 | -370.3 to 72.55 | ns | 0.3308 |
| 423-3E vs. 653-3E | -1415 | -1917 to -913.9 | **** | <0.0001 |
| 423-3E vs. 269-2F | -70.9 | -312.5 to 170.7 | ns | 0.9254 |
| 423-3E vs. 720-1E | -98.62 | -281.1 to 83.84 | ns | 0.5266 |
| 423-3E vs. 440-1E | -413.6 | -749.7 to -77.48 | * | 0.012 |
| 560-2F vs. 653-3E | -1266 | -1765 to -767.7 | **** | <0.0001 |
| 560-2F vs. 269-2F | 77.96 | -155.1 to 311.0 | ns | 0.8685 |
| 560-2F vs. 720-1E | 50.24 | -117.0 to 217.5 | ns | 0.9163 |
| 560-2F vs. 440-1E | -264.7 | -595.5 to 66.08 | ns | 0.1547 |
| 653-3E vs. 269-2F | 1344 | 842.2 to 1846 | **** | <0.0001 |
| 653-3E vs. 720-1E | 1317 | 825.4 to 1808 | **** | <0.0001 |
| 653-3E vs. 440-1E | 1002 | 466.6 to 1537 | *** | 0.0002 |
| 269-2F vs. 720-1E | -27.72 | -242.0 to 186.6 | ns | 0.9941 |
| 269-2F vs. 440-1E | -342.7 | -682.0 to -3.316 | * | 0.0472 |
| 720-1E vs. 440-1E | -315 | -631.7 to 1.797 | ns | 0.0515 |
| D7 | | | | |
| 423-3E vs. 560-2F | -527.3 | -1152 to 97.74 | ns | 0.1209 |
| 423-3E vs. 653-3E | -1088 | -1336 to -839.2 | **** | <0.0001 |
| 423-3E vs. 269-2F | -309 | -674.6 to 56.56 | ns | 0.1179 |
| 423-3E vs. 720-1E | 80.39 | -130.5 to 291.3 | ns | 0.8311 |
| 423-3E vs. 440-1E | -276.1 | -511.5 to -40.65 | * | 0.0156 |
| 560-2F vs. 653-3E | -560.4 | -1185 to 63.99 | ns | 0.0896 |
| 560-2F vs. 269-2F | 218.3 | -435.4 to 871.9 | ns | 0.8835 |
| 560-2F vs. 720-1E | 607.6 | -9.875 to 1225 | ns | 0.0547 |
| 560-2F vs. 440-1E | 251.2 | -370.6 to 872.9 | ns | 0.7559 |
| 653-3E vs. 269-2F | 778.6 | 413.8 to 1143 | *** | 0.0001 |
| 653-3E vs. 720-1E | 1168 | 958.7 to 1377 | **** | <0.0001 |
| 653-3E vs. 440-1E | 811.5 | 577.8 to 1045 | **** | <0.0001 |
| 269-2F vs. 720-1E | 389.4 | 36.42 to 742.4 | * | 0.0297 |
| 269-2F vs. 440-1E | 32.91 | -327.0 to 392.8 | ns | 0.9995 |
| 720-1E vs. 440-1E | -356.5 | -548.3 to -164.7 | *** | 0.0002 |
| D14 | | | | |
| 423-3E vs. 560-2F | -247.8 | -484.1 to -11.50 | * | 0.0362 |
| 423-3E vs. 653-3E | -1024 | -1258 to -789.0 | **** | <0.0001 |
| 423-3E vs. 269-2F | -252.4 | -549.6 to 44.73 | ns | 0.1163 |
| 423-3E vs. 720-1E | -3.71 | -220.7 to 213.3 | ns | >0.9999 |
| 423-3E vs. 440-1E | -516.1 | -1757 to 724.9 | ns | 0.6918 |
| 560-2F vs. 653-3E | -775.9 | -1033 to -519.0 | **** | <0.0001 |
| 560-2F vs. 269-2F | -4.613 | -315.5 to 306.3 | ns | >0.9999 |
| 560-2F vs. 720-1E | 244.1 | 1.876 to 486.3 | * | 0.0475 |
| 560-2F vs. 440-1E | -268.2 | -1510 to 973.6 | ns | 0.9685 |
| 653-3E vs. 269-2F | 771.2 | 461.3 to 1081 | **** | <0.0001 |
| 653-3E vs. 720-1E | 1020 | 779.4 to 1261 | **** | <0.0001 |
| 653-3E vs. 440-1E | 507.6 | -734.1 to 1749 | ns | 0.7079 |
| 269-2F vs. 720-1E | 248.7 | -51.97 to 549.4 | ns | 0.1319 |
| 269-2F vs. 440-1E | -263.6 | -1508 to 980.6 | ns | 0.972 |
| 720-1E vs. 440-1E | -512.4 | -1753 to 728.8 | ns | 0.6984 |
| D21 | | | | |
| 423-3E vs. 560-2F | -418.5 | -603.3 to -233.6 | **** | <0.0001 |
| 423-3E vs. 653-3E | -543.2 | -845.5 to -240.8 | *** | 0.0003 |
| 423-3E vs. 269-2F | -186.4 | -396.9 to 24.07 | ns | 0.1004 |
| 423-3E vs. 720-1E | -36.58 | -210.7 to 137.6 | ns | 0.9848 |
| 423-3E vs. 440-1E | 19.6 | -140.4 to 179.6 | ns | 0.9986 |
| 560-2F vs. 653-3E | -124.7 | -419.5 to 170.1 | ns | 0.7348 |
| 560-2F vs. 269-2F | 232.1 | 35.46 to 428.7 | * | 0.0166 |
| 560-2F vs. 720-1E | 381.9 | 228.7 to 535.1 | **** | <0.0001 |
| 560-2F vs. 440-1E | 438.1 | 302.5 to 573.7 | **** | <0.0001 |
| 653-3E vs. 269-2F | 356.8 | 50.91 to 662.6 | * | 0.0179 |
| 653-3E vs. 720-1E | 506.6 | 216.0 to 797.1 | *** | 0.0007 |
| 653-3E vs. 440-1E | 562.8 | 277.0 to 848.5 | *** | 0.0004 |
| 269-2F vs. 720-1E | 149.8 | -38.70 to 338.4 | ns | 0.1549 |
| 269-2F vs. 440-1E | 206 | 26.14 to 385.9 | * | 0.0239 |
| 720-1E vs. 440-1E | 56.18 | -61.38 to 173.7 | ns | 0.6375 |
| D28 | | | | |
| 423-3E vs. 560-2F | -199.1 | -368.2 to -30.12 | * | 0.015 |
| 423-3E vs. 653-3E | -385.6 | -706.4 to -64.72 | * | 0.0144 |
| 423-3E vs. 269-2F | 111.4 | -146.4 to 369.2 | ns | 0.7176 |
| 423-3E vs. 720-1E | -62.08 | -260.5 to 136.4 | ns | 0.9237 |
| 423-3E vs. 440-1E | 34.65 | -155.1 to 224.4 | ns | 0.9921 |
| 560-2F vs. 653-3E | -186.4 | -495.9 to 123.1 | ns | 0.3854 |
| 560-2F vs. 269-2F | 310.5 | 66.69 to 554.4 | * | 0.0121 |
| 560-2F vs. 720-1E | 137.1 | -31.16 to 305.3 | ns | 0.1517 |
| 560-2F vs. 440-1E | 233.8 | 76.44 to 391.2 | ** | 0.0022 |
| 653-3E vs. 269-2F | 497 | 149.1 to 844.9 | ** | 0.0032 |
| 653-3E vs. 720-1E | 323.5 | 3.265 to 643.7 | * | 0.047 |
| 653-3E vs. 440-1E | 420.2 | 103.6 to 736.8 | ** | 0.007 |
| 269-2F vs. 720-1E | -173.5 | -430.7 to 83.75 | ns | 0.2886 |
| 269-2F vs. 440-1E | -76.75 | -329.5 to 176.0 | ns | 0.9031 |
| 720-1E vs. 440-1E | 96.73 | -92.08 to 285.5 | ns | 0.6015 |
| D63 | | | | |
| 423-3E vs. 560-2F | 136 | 64.69 to 207.3 | **** | <0.0001 |
| 423-3E vs. 653-3E | 19.38 | -119.6 to 158.4 | ns | 0.9967 |
| 423-3E vs. 269-2F | -92.09 | -209.2 to 25.00 | ns | 0.153 |
| 423-3E vs. 720-1E | -76.47 | -186.6 to 33.67 | ns | 0.2737 |
| 423-3E vs. 440-1E | 17.7 | -113.3 to 148.7 | ns | 0.9969 |
| 560-2F vs. 653-3E | -116.6 | -257.5 to 24.32 | ns | 0.1335 |
| 560-2F vs. 269-2F | -228.1 | -347.2 to -108.9 | *** | 0.0004 |
| 560-2F vs. 720-1E | -212.4 | -325.6 to -99.31 | *** | 0.0002 |
| 560-2F vs. 440-1E | -118.3 | -251.3 to 14.72 | ns | 0.0939 |
| 653-3E vs. 269-2F | -111.5 | -270.5 to 47.52 | ns | 0.2693 |
| 653-3E vs. 720-1E | -95.85 | -253.0 to 61.26 | ns | 0.4184 |
| 653-3E vs. 440-1E | -1.675 | -170.1 to 166.7 | ns | >0.9999 |
| 269-2F vs. 720-1E | 15.62 | -123.1 to 154.3 | ns | 0.9991 |
| 269-2F vs. 440-1E | 109.8 | -42.72 to 262.3 | ns | 0.2422 |
| 720-1E vs. 440-1E | 94.18 | -56.11 to 244.5 | ns | 0.3874 |
